# Postgraduate student perceptions of face-to-face and distance education in orthodontics: A cross-sectional qualitative study

**DOI:** 10.1177/14653125221077108

**Published:** 2022-03-18

**Authors:** Olivia Johnson King, Fiona Ryan, Susan Cunningham

**Affiliations:** University College London Eastman Dental Institute, London, UK

**Keywords:** distance education, face-to-face education, orthodontic education, postgraduate student

## Abstract

**Objective:**

To investigate postgraduate student perceptions of face-to-face and distance education on a three-year programme in orthodontics.

**Design:**

Cross-sectional qualitative study.

**Setting:**

UCL Eastman Dental Institute, London.

**Participants:**

A total of 25 current postgraduate orthodontic students in the first, second and third years of training were included in this study.

**Methods:**

Postgraduate student perceptions were obtained by conducting online focus groups on Zoom Video Communications Inc. A focus group topic guide was developed, and a facilitator was trained to host the focus groups. There were separate focus groups for each year group, with a maximum of five participants in each group. The focus groups were audio recorded and transcribed verbatim. The transcripts were assessed by all members of the research team and analysed using a thematic content analysis, with a framework approach to identify themes and subthemes regarding perceptions of distance and face-to-face education.

**Results:**

A total of 25 students participated. Six key themes were identified relating to student perceptions of face-to-face and distance education: (1) social support network; (2) technology; (3) learning experience; (4) education environment; (5) interpersonal interactions; and (6) effective teaching/learning. There were perceived benefits and drawbacks for both modes of teaching delivery. In particular, students highlighted the importance of reliable technology, peer support and accessibility of educational resources for their academic learning. Students favoured a blended approach to learning where practical skills were taught in person and some theoretical aspects taught remotely.

**Conclusion:**

The results aid the understanding of how educational tools and digital technology can enrich the student academic experience. The results provide important information for the future development and delivery of orthodontic postgraduate education.

## Introduction

The COVID-19 pandemic caused significant disruption to educational systems worldwide, resulting in closures of schools, colleges and higher education institutions. Due to the need to control the spread of coronavirus, these institutions had to rely increasingly and, in some cases, exclusively, on digital tools and remote methods of learning in a synchronous and asynchronous manner to deliver their educational programmes. With the suspension of face-to-face teaching and resultant changes to the methods of teaching delivery, it was important that the quality of student education was maintained and impacts on academic learning minimised. There was an onus on educators to adapt rapidly to ensure that they provided the necessary information to students in an online format without compromising their teaching standards or quality of teaching.

With regard to orthodontics, educators have traditionally relied on face-to-face lectures in a didactic format (Chadwick et al., 2002). Interestingly, Rao and colleagues (2020) conducted a systematic review to investigate the use of e-learning in orthodontic graduate education and noted that with newer advances in technology and students who are ‘digital natives’, methods of knowledge delivery need to be reassessed in orthodontic training. The COVID-19 pandemic has undoubtably provided a catalyst for the restructuring of educational systems and, going forward, it is essential that this momentum is sustained to ensure that the new educational model is of an optimal standard for students.

There have been limited studies that have investigated student perspectives of distance education in postgraduate dentistry compared with face-to-face education (Kunin et al., 2014; Rosenbaum et al., 2012). At present, most of the research in this emerging area is quantitative and questionnaire-based and does not provide a full understanding of the reasons behind the perceptions about different modes of teaching (Dost et al., 2020).

It is therefore imperative to investigate this area to aid understanding and improve the delivery of orthodontic programmes for students. In addition, research in this area will help aid the understanding of how educational tools and digital technology can be used to enrich the student experience and will provide invaluable information to educational institutions, to help shape the future development of orthodontic postgraduate education both nationally and internationally.

The aim of this study was to investigate postgraduate student perceptions of face-to-face and distance education in orthodontics and to determine the most effective and engaging methods of teaching to provide students with an optimal education experience.

## Material and methods

This qualitative study investigated postgraduate student perceptions of face-to-face and distance education in orthodontics at one UK dental school. Ethical approval was granted by University College London (UCL) Research Ethics Committee (18239/001) and the project was registered with the UCL Data Protection Office. A Data Sharing Agreement was signed with a professional transcription company to meet GDPR requirements.

Participants were recruited from the Orthodontic Department in a large postgraduate teaching hospital in the United Kingdom. Before commencing recruitment, the study was introduced during departmental staff meetings and it was explained that potential participants would be contacted individually by the research team to ask if they would be happy to consider participating in the study; however, it was stressed that participation was voluntary and they did not have to take part if they did not feel comfortable doing so. Anyone who did not wish to be contacted in this way was asked to let the lead investigator know. Individual invitation emails were subsequently sent to all postgraduate orthodontic students (n=30) on the three-year programme of study who had received both face-to-face and distance education between March 2020 and January 2021; email addresses were available from the existing departmental staff/postgraduate lists. The aim was to include students from all three year groups, representing different genders and national/international educational backgrounds. All potential participants were asked to respond to the email to confirm whether they were happy to take part in the study. In total, 25 students agreed to participate in the focus groups; there were five students who were unable to participate due to illness scheduling conflicts or time zone differences. After confirmation of their wish to participate, participants were asked to complete and return a signed consent form.

Postgraduate student perceptions were investigated through online focus groups on Zoom Video Communications, Inc. (Zoom) and facilitated by the researcher (OJK) who underwent focus group facilitation training and undertook practice interviews with the senior researchers. The researchers felt that there were potential benefits to using an ‘insider’ facilitator for the focus groups; the facilitator had thorough knowledge of the academic programme, an ability to draw on experience when probing during the focus groups, and there was a willingness and openness of participants to discuss issues with a familiar facilitator, which optimised the data yield.

A topic guide was developed by the project investigators to guide the discussions in the focus groups, explore perceptions of face-to-face and distance education, and to ensure adequate coverage of key topics. An initial draft of the topic guide was developed based on a thorough review of the literature in this area as well as based on the aims of the study and the aspects of orthodontic education, which the researchers intended to explore. The topic guide commenced with an introduction to set the context for the focus group discussion and consisted of four sections with questions about traditional face-to-face education, distance education, a comparison of the two teaching methods and assessments. Following the practice interviews, the topic guide was amended and during the focus group interview process the guide was modified if previously unidentified, but pertinent, topics emerged. The topic guide was not rigidly followed in this order, but all aspects were covered, and the order adapted according to the flow of the focus groups.

Participants were grouped according to their year of training to ensure they felt comfortable in the focus group environment. The focus groups were recorded locally on Zoom and the audio recordings were transcribed by a professional transcription company with any identifiers removed. There were seven focus groups and the group sizes were in the range of 3–5 participants, as the researchers felt this was a reasonable number of participants to manage in an online focus group.

A content thematic analysis using the Framework method was used to analyse the data, based on the methodology developed by the National Centre for Social Research (Ritchie and Spencer, 1994). The identification of themes and subthemes was conducted by three researchers (OJK, FR, SJC) independently; all three researchers read and reread the transcripts and agreed on the themes and subthemes. Each of the themes were colour-coded and grouped to allow for comparison and checking. Quotes from the transcripts were inserted into Microsoft Excel; each theme was allocated a separate worksheet and the column represented the subthemes. Each student was allocated a row and the relevant quote from the transcripts was entered in the relevant cell in Excel; this method allows researchers to track findings, helping ensure that links between the original data and findings are maintained and transparent.

## Results

In total, 25 postgraduate students were recruited, including 15 UK and 10 international trainees, representing 83% of trainees on the programme (Table 1). The timing of the focus groups was such that it was possible to include two cohorts of third-year students: those who finished their orthodontic training in summer 2020 and those completing training in summer 2021. The focus group interviews lasted 51–81 minutes.

**Table 1.** Demonstrating student participants according to year group.

Year group	No. of trainees	UK students	International students	Year of training completion
1	8	6	2	2023
2	7	3	4	2022
3	6	3	3	2021
3	4	4	0	2020

From the analysis, six themes were identified and within each theme there were several subthemes (Table 2). The results are presented using direct verbatim quotes from participants (e.g. P1 = participant 1) to support the identified themes and subthemes.

**Table 2.** Demonstrating the main themes and subthemes from the transcripts.

Main themes	Subthemes			
Social support network	Bonding with peers	Peer support	Social interaction	
Technology	Unpredictability of technology	Hardware and software requirements	Financial implications	
Experience	Accessibility	Convenience	Diversity (of educator pool, of location)	Efficiency
Education environment	Familiarity	Setting		
Effective teaching and learning	Peer learning	Practical skills learning	Theoretical learning	
Interactions	Exposure (can lead to feelings of vulnerability)	Physical presence	Immediacy (engagement, interactions, practical skills)	Impact on teacher-student relationship

### Social support network

This theme related to the importance of social interaction and bonding between students as part of their educational experience. There were three subthemes identified.

#### Bonding with peers

Students explained that the distance education format made it more difficult to connect with their peers and build rapport as they could not get to know each other as well.

‘*The disadvantage of distance learning is that you don’t get to actually speak to people, so you don’t get to know them as well.*’ (P2)

#### Peer support

Students explained that peer support provided benefits such as being able to have informal discussions and ‘bouncing ideas’ off each other.

‘It’s *really beneficial to have face-to-face teaching, see my peers and have informal discussions. When we’re doing Zoom teaching, we don’t really get that opportunity.*’ (P12)

#### Social interaction

Students discussed the difficulty of socialising online and some felt that the distance education format was a hindrance to social interaction.

‘*I think it’s important to have that social element to the teaching which you totally lose from it being online.*’ (P23)

### Technology

In this theme, some students discussed the negative impacts of technology on their education as it could sometimes be unpredictable; there were no positive aspects discussed in relation to technology. There were three subthemes.

#### Unpredictability of technology

The unpredictability of technology was highlighted as a significant disadvantage of distance education. The problems raised were either related to the instability of Internet connections or laptop problems, both of which affected the quality of distance teaching sessions.

‘*One thing we experienced during these online lectures is that the quality of teaching always depends on the quality of the Internet connection.*’ (P20)

#### Hardware and software requirements

Some students explained that they needed to upgrade their hardware and software to facilitate learning in a distance format.

*“You need to make sure you’ve got the correct apps and correct technology in order to do virtual learning”*

#### Financial implications

Some students reported a financial burden, as they had to invest in new equipment in order to participate effectively in online teaching.

‘*My laptop was slow anyway, so it doesn’t matter, but I forked out two thousand pounds to buy a new laptop.*’ (P25)

### Experience

There were four subthemes that were identified within this theme.

#### Accessibility

Accessibility of materials and resources was an important subtheme that arose. Students noted that a key benefit of distance education was being able to access educational material at any time.

‘*I love that we have the recordings, and we can access them whenever we want, we can make notes at our convenience, I really love having the theory part online.*’ (P8)

#### Convenience

One perceived downside of face-to-face education was the need to commute in order to access the education, making this form of teaching less convenient.

‘*Commuting is a bit of an issue with face-to-face teaching, especially if you’ve got multiple trains you have to take.*’ (P12)

#### Diversity

Distance education also allowed for diversity of the educator pool; some students explained that online teaching provides an opportunity to get better access to external lecturers.

‘*Now we’re doing things online, there’s more of an opportunity to get external lecturers, even if it’s from other UK hospitals.*’ (P12)

#### Efficiency

Several students expressed that they found distance education more efficient than face-to-face teaching as they did not have to waste time travelling and therefore had more time to prepare for teaching sessions.

‘*I also feel like I have more time to prepare because I’m not travelling.*’ (P4)

### Education environment

The environment in which students were receiving education also arose as an important theme and there were two subthemes that arose within this theme.

#### Familiarity

Some students commented on the benefits of being able to study in familiar environments such as their own home; they explained that learning from home provided a more comfortable environment.

‘*When you’re in the comfort of your own home you can actually kind of take breaks and digest a bit more.*’ (P25)

#### Setting

For some students, being in a formal teaching setting such as a lecture theatre was beneficial for their concentration but, in contrast, others found it beneficial to be doing online teaching at home as this improved their concentration.

‘*I know it’s cosy at home but sometimes I just can’t be bothered to log into Zoom, whereas if I’m sat in a lecture theatre it just feels like I’m there to learn rather than just be at home.*’ (P4)

*‘I find that I concentrate better when it’s online teaching, I think it’s because I’m just looking at the screen and there’s no distractions.*’ (P10)

### Effective teaching and learning

In this theme students expressed the importance of learning from their peers, the limitations of learning practical skills online and the benefits of learning theoretical content in a distance format.

#### Peer learning

Students highlighted that learning from their peers was an important part of the education process and some felt that face-to-face education was better at facilitating peer learning than distance education.

‘*I think face-to-face teaching is easier for peer-to-peer learning because you can ask colleagues’ advice straightaway.*’ (P10)

#### Practical skills learning

Students across all three year groups discussed the difficulty of learning practical skills in a distance format and strongly felt that orthodontic practical skills teaching had to be face-to-face.

‘*It’s the hindrance that online teaching presents itself…you can’t really assess practical skills, you can’t really develop practical skills.*’ (P18)

#### Theoretical learning

In general, students felt that theoretical and didactic teaching was taught well in a distance format and felt it was equally as effective as theoretical face-to-face teaching.

‘*All the theoretical ones that we’ve had so far, I’m more than happy with them online, actually I’m more happy with the online than I am with the face-to-face.*’ (P7)

### Interactions

Students discussed the importance of human interactions in their educational experience and there were four subthemes identified within this theme.

#### Exposure

Some students said they felt more self-conscious and less ‘comfortable’ being online as they felt more exposed, especially if a teaching session was being recorded. Due to this, they were less likely to ask questions during an online teaching episode.

‘*If I was really struggling with a topic, I don’t know how comfortable I would feel to bring that up with anybody in a teaching session virtually.*’ (P22)

#### Physical presence

Several students commented on the importance of physical presence in the education experience; students felt it was easier to interpret the body language of educators and fellow students when in a face-to-face setting.

‘*It’s easier to ask questions face-to-face than it is online, because you’re maintaining eye contact with the teacher, the teacher can gauge when you don’t quite understand something.*’ (P2)

#### Immediacy

Interestingly, students commented on the differences in engagement and interaction with the different teaching methods and some students noted that they were less interactive online.

‘*For me it doesn’t really work very well online because you don’t get that immediate feedback from people and people are less likely to discuss as we would face-to-face.*’ (P3)

#### Impact on teacher–student relationship

In general, students felt that face-to-face teaching was beneficial for nurturing the teacher–student relationship as it made them feel more connected to educators.

‘*I prefer face-to-face because the environment is more encouraging when it comes to the teacher/student relationship.*’ (P5)

## Preferred teaching methods

At the end of each focus group, each participant was asked which teaching method they preferred: face-to-face, blended or distance learning; the majority of students expressed a preference for a blended approach and wanted this method to be used for the orthodontic programme going forwards. Participants commented that they found distance teaching more time efficient and it allowed them more time to prepare for teaching sessions as they did not need to travel to access that teaching. The majority of students expressed that they wanted the online format of teaching to continue for theoretical orthodontic topics; however, they felt strongly that practical skills needed to be taught using a face-to-face approach. These findings highlighted the benefits of a blended approach and a hybrid teaching model, utilising the benefits of both modes of teaching delivery to provide an optimal learning experience for students.

## Discussion

The COVID-19 pandemic has been a catalyst for the inevitable cultural transformation of the educational system and the circumstances have given rise to an enhanced hybrid model of education involving face-to-face and distance education. As this model of education has developed, going forward it is crucial to determine the best teaching methods to improve curricula and to maximise the benefits of both face-to-face and distance education. Thus far, there has been limited research to assess student perceptions of distance and face-to-face teaching in orthodontics and there is a clear need to conduct research in this area. This study therefore adds to the evidence base in this area.

Six themes were identified from the analysis of the focus groups. With regard to the first theme, social support network, students explained that the distance education format made it more difficult for them to bond with their peers and they could not get to know each other as well. Harasim and colleagues (1995) found that the establishment of social bonds has important cognitive and socio-effective benefits for learning. Interestingly, Alqurashi (2019) found that socialising and interactions between students had a positive impact on student satisfaction and the perceived effectiveness of online learning. The present study supports previous findings that social interaction is an important aspect in the delivery of education and is easier to achieve in a face-to-face format.

Technology was the second theme and, within this, students reported that technology may be unreliable which could negatively impact on their education where distance teaching was being provided. This was also experienced by undergraduate dental students in Indonesia in a study by Amir et al. (2020). In addition, research found that an unstable Internet connection was a barrier to distance learning for 21.53% of UK medical students (Dost et al., 2020). In the present study, students across all three year groups discussed the benefits of being able to access pre-recorded lectures online as it allowed them to review educational material at the time of their choosing. Recording lectures and maintaining their availability online may therefore help counteract any technological difficulties faced by students during online synchronous sessions; however, this issue clearly remains one of the real limitations of distance learning.

The educational experience was discussed in relation to accessibility of educational resources, the convenience of accessing education without commuting, diversity of the educator pool and time efficiency. A reported benefit of distance education was students being able to access and review educational material at a time of their choosing. A reported strength of the virtual learning environment for orthodontic postgraduate students in a previous study also included the improved access to resources and ability to interact with the programme regardless of distance (Shah and Cunningham, 2009). These findings were also supported by Rad and co-workers (2021) who reported that online education made resources more accessible for dental students.

In the ‘education environment’ theme, there was no clear consensus about the best education setting. For some students, working from home in a distance format was beneficial for their concentration and for others face-to-face teaching in a traditional lecture setting improved their concentration. In the literature, there is also no clear consensus as individual circumstances and preferences clearly play a key role (Grimes, 2002; Rad et al., 2021); and it is likely that the education setting will always pose a challenge for educators, and this could be the reason why blended courses where there is a combination of face-to-face and distance education are viewed favourably by students.

In the ‘effective teaching and learning’ theme, students discussed peer learning, practical skills teaching and theoretical teaching. In general, students felt that peer learning was facilitated in a face-to-face setting. The consensus from students with regard to practical skills was that these could only be taught effectively face-to-face. This is supported by other studies investigating medical and dental student perspectives on distance and face-to-face learning (Dost et al., 2020; Wang et al., 2021). In the present study, the majority of students expressed that they would like the online format of teaching to continue for theoretical orthodontic topics and replace face-to-face teaching in these areas; this finding was also similar to the study by Schlenz et al. (2020) who found that dental students wanted approximately half of their theoretical teaching to be online in the future.

In the final theme, interactions, several students expressed that online teaching could make them feel exposed and vulnerable, which prevented some from asking questions during the teaching session. Some students found they were less interactive online, and this format made them feel disconnected; this highlights the importance of ensuring educators are aware of this and are able to manage these aspects of teaching with sensitivity. Grimes (2002) also reported that the face-to-face classroom allowed the discussion of specific student questions, but this was not as easy in a distance format. In addition, Grimes found that 69% of dental assistants and dental hygiene students reported feeling detached from the faculty and other students with distance education. Varvara and co-workers (2021) noted that dental students found interactivity challenging with distance education as there was little possibility for discussion. Students in the present study found that face-to-face teaching was beneficial to nurture a relationship between students and educators. McCann et al. (2010) concluded that although students found that electronic materials can enhance learning, they wanted face-to-face contact with faculty and expressed that electronic resources should not replace faculty interaction.

The present study has the advantage of having included both UK-based and international orthodontic trainees in order to ensure a breadth of opinions, which enhanced generalisability and wider transferability of results. In addition, students were recruited from all three year groups in order to obtain opinions from those at different stages of training. The authors acknowledged that students from the different year groups had varying levels of experience with asynchronous and synchronous teaching methods on the programme. Despite these differences, the authors believe that obtaining opinions from students at various stages of training provided a greater diversity of opinions and optimised the data yielded.

As this study was carried out at one postgraduate dental school, the authors are aware that the results may not be applicable to all orthodontic students nationally and internationally. Future research could therefore be conducted at other dental institutions, including larger cohorts of students, to obtain a broader cross-section of views and make results more generalisable.

The researchers made a decision to use focus groups rather than one-to-one interviews for this study; prior research has found that focus groups allow for a greater range of discussion between the participants (Schneider et al., 2012). In addition, the interaction between participants in focus groups generates accounts which may be more fully articulated and detailed than one-to-one interviews (Wilkinson, 1998). The reported optimal number of participants for a focus group varies; however, Krueger and Casey (2000) suggested that smaller groups of 6–8 participants have greater potential. For this study, the focus group sizes were in the range of 3–5 participants as the researchers felt this would be a reasonable number of participants to manage in an online focus group. As each year group contained 6–9 students, year groups were subdivided into smaller focus groups for participation in the study. These smaller groups also allowed the researcher (OJK) to accommodate variations in individual student timetables. The focus groups took place at a time when students were only receiving distance education due to the national restrictions on face-to-face education with the COVID-19 pandemic. It is important to note that the timing of these focus groups may have impacted on the data collected as the students were not able to have simultaneous face-to-face and distance teaching and therefore it was not possible to have a concurrent comparison of the two teaching methods. However, all participants had wide prior experience of face-to-face teaching with which to make comparisons.

The researchers considered whether to utilise an ‘outsider’ or an ‘insider’ as a facilitator of the focus groups. Insider research studies have been criticised for the research being ‘too close’ for objectivity (Brannick and Coghlan, 2007). However, this type of research also has multiple benefits, including having perspective of the culture of the academic programme, ability to draw on understanding and experience when probing during the focus groups, and the willingness of participants to discuss issues with someone who understands them (Fleming, 2018). The challenges of insider research include inherent subjectivity, the potential for implicit coercion of participants, potential of professional conflicts and researcher bias (Fleming, 2018). After considering the advantages and disadvantages, the researchers felt that the benefits of ‘insider’ facilitator knowledge of the programme structure and participants outweighed the perceived disadvantages. To minimise any disadvantages of insider research, the researcher OJK underwent focus group interview training during which emphasis was put upon remaining neutral and impartial and avoiding the temptation to share experiences and introduce bias.

The COVID-19 pandemic has provided an opportunity for cultural transformation of the educational system and the present circumstances are likely to give rise to further developments of the hybrid model of education to maximise the benefits of both face-to-face and distance education. In light of the findings in this study, the authors would recommend the following:

- Educators should ensure a blended learning approach for orthodontic programmes; with practical components/skills being taught in a face-to-face setting but with some of the more theoretical aspects of teaching delivered remotely.
- Online libraries of pre-recorded lectures and practical skills videos should be expanded and made available for all students (e.g. via local virtual learning environments or potentially through the British Orthodontic Society Virtual Learning Environment).

## Conclusion

This research aids the understanding of how students perceive face-to-face and distance education in orthodontics and the results provide valuable information for the future development of orthodontic education.

Although there were challenges noted with distance education, the majority of students felt that blended learning would be their preferred way to engage with the orthodontic curriculum. Several students felt that some of the theoretical orthodontic subjects could be delivered effectively in a distance format and would like this format to be used going forward but more practical elements of the curriculum should continue to be delivered in a face-to-face format.

## Supplemental Material

sj-docx-1-joo-10.1177_14653125221077108 – Supplemental material for Postgraduate student perceptions of face-to-face and distance education in orthodontics: A cross-sectional qualitative study Click here for additional data file. Supplemental material, sj-docx-1-joo-10.1177_14653125221077108 for Postgraduate student perceptions of face-to-face and distance education in orthodontics: A cross-sectional qualitative study by Olivia Johnson King, Fiona Ryan and Susan Cunningham in Journal of Orthodontics
