# Supplementary material for: Postgraduate student perceptions of face-to-face and distance education in orthodontics: A cross-sectional qualitative study
Source: J Orthod. 2022 Mar 18;49(3):280–7. doi: 10.1177/14653125221077108 (PMC9421199; doi:10.1177/14653125221077108)
Supplement: sj-docx-1-joo-10.1177_14653125221077108 – Supplemental material for Postgraduate student perceptions of face-to-face and distance education in orthodontics: A cross-sectional qualitative study [file sj-docx-1-joo-10.1177_14653125221077108.docx]

**Topic Guide for Student Focus Groups**

**Project Title: Postgraduate student perceptions of face-to-face and distance education in Orthodontics: a cross-sectional qualitative study**

**Aims and Objectives:**

- To ascertain the perceptions of postgraduate orthodontic students regarding traditional face to face education
- To ascertain the perceptions of postgraduate orthodontic students regarding distance education
- To compare and contrast perceptions of both traditional face-to-face and distance education
- To ascertain preferences of postgraduate orthodontic students when considering traditional face-to-face versus distance education

1. **Introduction:**

*Aim: to introduce the topic of the focus group and set the context for the proceeding discussion.*

- Introduce self as facilitator
- Introduce study: aims and overview
- Explain and define key terms: face-to-face and distance education:
  - Face-to-face education defined as *“education in a physical setting specifically dedicated to learning activities, the presence of a professional teacher, books and other learning materials”* (Cropley and Kahl 2011).
  - Distance education defined as *“all arrangements for providing instruction through print or electronic communications media to persons engaged in planned learning in a place or time different from that of the instructor or instructors”* (Moore, 1990).
- Key points:
  - - - Purpose of study
      - Audio recording
      - Confidentiality
      - Duration of focus groups
      - Voluntary nature of participation
      - Right to withdraw
      - Data and publication
      - Any questions?

1. **Dental degree and current year of study:**

*Aim: to ascertain when participants graduated from dental school and from where.*

- In which country did you undertake your undergraduate dental training and which year did you graduate?
- What year of study are you currently in?

1. **Traditional face-to-face teaching/education:**

*Aim: to ascertain perceptions of face-to-face teaching (e.g. classroom synchronous teaching episodes like lectures, case seminars, journal clubs etc.)*

- As a postgraduate, what do you think are the benefits of face-to-face teaching/education?
- What do you think are the drawbacks/limitations of face-to-face teaching/education?
- What subjects/topic areas do you think are best suited to face-to-face teaching/education?
- Do you think there is still a place for face-to-face teaching in orthodontics?

1. **Distance teaching/education:**

*Aim: to ascertain perceptions of distance teaching (e.g. lectures/seminars/journal clubs, usually conducted via electronic communications where participants are in a separate time or place from each other)*

- As a postgraduate, what do you think are the benefits of distance teaching/education?
- What do you think are the drawbacks/limitations of distance teaching/education?
- Do you think these benefits and drawbacks apply routinely or were due to the urgent introduction during COVID-19?
- What subjects/topic areas are best suited to distance education?
- How do you think distance teaching could be used most effectively in orthodontics moving forwards?

1. **Comparison of face-to-face and distance teaching/education:**

*Aim: to compare and contrast* face-to-face *and distance teaching/education and ascertain preferences.*

- Do you prefer face-to-face or distance teaching/education?
- Do you feel that one method is superior to the other?
- How would you like to see the two methods of teaching used in the future so that you still have an effective teaching experience?

1. **Assessments:**

*Aim: to ascertain experiences of distance assessments.*

- For those who have had/undergone remote assessments, how did you feel it worked (considering different assessments separately e.g. written papers, vivas)
- What do you think the benefits were? What were the drawbacks/limitations?
- Would you have a preference for one method of assessment over the other? (again thinking of different types of assessments individually)
